# Should Liver Transplants be Performed for Malignant Disease?

**DOI:** 10.1155/1989/17497

**Published:** 1989

**Authors:** K. E. F. Hobbs, Henry A. Pitt

**Affiliations:** 1 Royal Free Hospital School of Medicine Pond Street London NW3 2QG UK; 2 Blalock 688 Johns Hopkins Hospital 600 N. Wolfe Street Baltimore MD USA


*HPB Surgery* 1989, Vol. 1, pp. 239–256
Reprints available directly from the publisher
Photocopying permitted by license only

© 1989 Harwood Academic Publishers GmbH
Printed in the United Kingdom

### HPB INTERNATIONAL

**EDITORIAL & ABSTRACTING SERVICE** JOHN TERBLANCHE, EDITOR
Department of Surgery,
Medical School · Observatory 7925
Cape Town · South Africa
Telephone: Department Office: 47-1250
Hospital Connection: 47-3311
Telex: 5-22208
Tel. Add: ALUMNI CAPETOWN
Telefax: (021) 47-8955

### SHOULD LIVER TRANSPLANTS BE PERFORMED FOR MALIGNANT DISEASE?

### ABSTRACT

*O'Grady, J.G., Polson, R.J., Rolles, K., Calne, R.Y., Williams, R.* (1988) Liver Transplantation for Malignant Disease: Results in 93 consecutive patients. Annals of Surgery, 207, 373–9.

**Ninety-three patients with malignant disease underwent orthotopic liver transplantation between May 1968 and April 1987 in the Cambridge/King's Hospital program. Of 50 patients with primary hepatocellular carcinoma (HCC) (19 with cirrhosis, 31 without cirrhosis, including 7 with fibrolamellar variant), 37 (74%) survived for more than 3 months, and in this group evidence of tumor recurrence was obtained in 24 (64.9%), the longest survivor being 11.8 years post-transplant, and three survived for more than 5 years. Although there is no correlation between the frequency of tumor recurrence and underlying cirrhosis, or histologic type (except fibrolamellar variant), it was observed earlier in those with moderate/poorly differentiated tumors and also when prednisolone and azathioprine was used for immunosuppression. Tumor recurred in all but two of those with peripheral or central cholangiocarcinoma (one alive at 6.1 years) with median survival times of 34 weeks and 56 weeks for the central and peripheral types, respectively. Among the unusual primary tumors, one with epithelioid haemangioendothelioma developed tumor recurrence at 2 years, one of two patients with apudoma is tumor-free at 2.2 years, and the one patient with bile-duct papillary cystadenocarcinoma is alive at 1.7 years. For the secondary hepatic malignancy group, survival times were shorter with little palliation except for two patients with carcinoid syndrome who were free.**

**DISCUSSION**

The traditional surgical ethos of cancer treatment is to obtain a cure by removing all malignant tissue. Primary liver cell cancer, often associated with cirrhois, is probably the world's commonest primary malignancy and secondary tumour deposits in the liver are frequently seen by all surgeons. In both cases surgeons argue that resection will lead to an improved prognosis. However hard data to prove this is difficult to find and some caution is needed before reaching this conclusion[1]. When tumour replacement of the liver parenchyma is so great that resection of all of it is impossible without removing the whole organ, transplantation should be the ideal approach. Is it?

Starzl's team has grave doubts arguing that the vast majority of such patients have recurrence of malignancy within one to four years of successful transplantation[2]. The longest survivors in their series are patients with the fibrolamellar varient of primary liver cell cancer and those in whom a cancer was found in the recipient's liver incidentally after transplantation had been carried out for some other reason[3]. Conversely Pichlmayr argues, 'But all these arguments (on value and justification) are somewhat problematic and should be negated if life saving prolongation in a good state of health could possibly by achieved'[4].

The team headed by Professor Roy Calne and Dr Roger Williams at King's College Hospital, London and Addenbrooke's Hospital, Cambridge, England now reports its results of 93 liver graft operations carried out for malignant disease of the liver. Twelve of 50 patients who had primary hepatocellular cancer survived for more than one year including two who were tumour free at death over five years after transplantation. Of the ten still alive, six are tumour free, one at 4.8 and one at 11.8 years. The presence of cirrhosis did not significantly affect the outcome. Of the seven patients with the fibrolamellar varient, three died with recurrence, one died at 5.8 years from transplant related problems but was tumour free and three are alive and tumour free at three months, 1.8 and 4.5 years.

Of 26 patients with cholangiocarcinoma, only one is alive and tumour free at 6.5 years. The median survival for patients with central cholangiocarcinoma was 34 weeks and with peripheral tumour 56 weeks. In a recent review Starzl's group lists this type of tumour as a 'relative contraindication' to transplantation[2]. However Pichlmayr claims a hundred per cent one year survival for 'proximal bile duct tumours — in lymphnode negative stages' but only 13% one year survival if lymphnodes were involved[4] and he argues that making the presence of bile duct tumours a contraindication to transplantation may be 'premature at the present.'

Two out of nine of the transplants for 'rare malignancies' are tumour free and well at 20 months and 2.2 years. The rest have succumbed.

Of the six patients who were transplanted for metastatic disease none survived more than 41 weeks. One of two patients transplanted for carcinoid syndrome remains symptom free at 12 months despite rising levels of 5 HIAA.

This well documented paper constitutes a major contribution to the debate but firm conclusions are still difficult to reach. At a simplistic level it would appear that if the malignant process is totally confined to the liver and it can be removed and replaced with a good new organ then cure is effected. However this is only occasionally possible. In most cases at the time of transplantation the tumour has already seeded outside the liver and it is these metastases which ultimately contribute to the patient's death. Certainly the morbidity and mortality of the surgical procedure is now improving.

Liver transplantation for malignant disease is an attractive surgical procedure since many patients (in the West) do not suffer concomitant cirrhosis (19 out of 50 in this study) and thus the operation is usally technically straightforward without the excessive haemorrhage associated with portal hypertension.

Can we identify the suitable candidates for this procedure? It would appear that patients with fibro lamellar varient of primary liver cell cancer and those with a proximal cholangiocarcinoma and no involved nodes fare best. It is difficult to advise transplantation for secondary tumours despite occasional reports of successful operations. The ideal patient will be one in whom resection of the tumour is impossible because of its extent but in whom there is no seeding outside the liver. Identification of these patients is difficult. Macroscopic tumours can be readily identified by conventional imaging such as ultrasound, CT or at laparotomy. However micrometastases cannot be found. We have to await further developments of targeted markers in the molecular biology laboratories before they can.

What should be done in the meantime? Liver transplantation is now an acceptable surgical operation and major centres will continue to practise it in an attempt to treat malignant disease affecting the liver. They should continue to do so but healthy, critical caution must be exercised when advising this treatment which is expensive in terms of money and patient discomfort and still does not carry any guarantee of success. Some patients with confined tumours will benefit and gain a few extra months or even years of life. The results of prospective controlled trials would be interesting but they are probably impossible to mount and anyway will probably fail to give any help in decision making for an individual patient. Hopefully further work in the basic science laboratories will bear fruit and allow us to select (the few) ideal candidates in the future.

**Keywords:** Transplantation, liver, liver tumour.

Professor K.E.F. Hobbs
Royal Free Hospital School of Medicine
Pond Street
London NW3 2QG
England

### REFERENCES

1. Editorial (1988) Resection of haematogenous metastases. Lancet ii, 485–487
2. Maddrey, W.C., Van Thiel, D.H. (1988) Liver transplantation: An overview. *Hepatology,* **8**, 948–959
3. Iwatsuki, S., Gordon, R.D., Shaw, B.W., Starzl, T.E. (1985) Role of liver transplantation in cancer therapy. *Annals of Surgery,* **202**, 401–407
4. Pichlmayr, R. (1988) Is there a place for liver grafting for malignancy? *Transplantation XX:Suppl 1;* 478–482

### DISCUSSION

Over 40 years ago, Mixter and his associates[1] demonstrated that cholangiovenous reflux occurs when contrast media is injected into the bile duct. Twenty years ago Huang *et al*[2] demonstrated in dogs that cholangiovenous reflux of *Escherichia coli* is directly related to biliary pressure. These investigators also showed that cholangiolymphatic reflux of bacteria occurs at the same levels of intrabiliary pressure which cause cholangiovenous reflux. In 1984 Yamamoto and Phillips[3] performed corrosion-cast experiments in rats and noted filling of periportal lymphatic spaces after biliary injection. One theoretical problem with this study, however, was the relatively high viscosity of the casting compound. Therefore, Stewart *et al*[4] have recently repeated these studies with a lower viscosity mixture which approximates the viscosity of bile.

The findings of Stewart *et al*[4] confirm the earlier observations of Yamamoto and Phillips[3]. These studies have demonstrated that cholangiovenous reflux in the rat progresses from the proximal bile ductules into 1) the spaces of Mall and Disse, 2) the hepatic sinusoids, and 3) the collecting veins. This process occurs without filling of bile canaliculi and without disruption of hepatocytes. Moreover, particles of 1.7 μ or smaller were able to reflux in this manner whereas particles of 10μ size did not reflux. This observation is consistent with previous studies which have demonstrated that bacteria can reflux into both the hepatic veins and lymphatics while erythrocytes, which are 6 or 7μ in diameter, are too large to reflux even at high pressures.

An interesting additional observation made by Stewart *et al*[4] was that the "resistance" to reflux varied as this process occurred progressively into 1) the spaces of Mall and Disse, 2) the hepatic sinusoids, and 3) the collecting veins. Resistance, as measured from pressure/volume relationships, increased as reflux progressed from the bile ductules into the spaces of Mall and Disse at pressures from 20 to 50 cm of water. Resistance then decreased to values observed during biliary ductal filling as reflux continued into the hepatic sinusoids at pressures from 50 to 80 cm of water. Resistance then fell to practically zero as reflux progressed into the hepatic collecting veins at pressures of approximately 80 cm of water.

These "resistance" data were obtained by retrograde injection into the common bile duct at a constant rate of 0.04 ml/min and correlated with electron microscopy. An interesting extension of these studies would be to measure resistance and observe reflux pathways at different flow rates. Clinically, cholangitis, and therefore reflux, is most likely to occur with rapid increases in intrabiliary pressure. The question remains, therefore, whether the reflex pathways observed by Stewart *et al*[4] would be altered by different flow rates. If the infusions had been more rapid, would the pathways have differed and would "high-resistance pathways" such as biliary canaliculi or hepatocytes have been involved?

Another interesting question is the relative contribution of cholangiovenous and cholangiolymphatic reflux to clinical eholangitis. The studies by Yamamoto and Phillips[3] and by Steward *et al*[4] both suggest that cholangiolymphatic reflux occurs before and at lower pressures than cholangiovenous reflux. This observation suggests that if intrabiliary pressures are raised to only moderate levels (20 to 50 cm of water) cholangiolymphatic reflux may be the only route for bacteria to gain access, via the thoracic duct, to the venous system. This scenario would be possible because at pressures below 50 cm of water Stewart *et al*[4] did not observe reflux into the hepatic sinusoids or collecting veins.

Another issue that must be considered is the relevance of these rat studies to man. Humans and many other species have a gallbladder, but the rat does not. One of the important functions of the gallbladder is to absorb water and, thereby, concentrate bile. In the face of distal biliary obstruction, the absorptive function of the gallbladder may actually moderate intrabiliary pressures and keep them below the hepatic secretary pressure. Thus, a species such as the rat, which does not have a gallbladder, may have reflux pathways that are different from those present in man. Conformation of the studies by Stewart *et al*[4] in a species with a gallbladder would add credence to their observations.

A third point that must be considered when interpreting these corrosion casting studies is the similarity of the casting compound to bile. Stewart *et al*[4] have attempted to improve upon the study by Yamamoto and Phillips[3] by lowering the viscosity of the casting compound. Stewart and her colleagues demonstrated quite nicely that the addition of inert ceramic particles of different sizes dramatically affected "reflux pathways." What would they have observed, however, had they also studied the effect of varying the osmolality or pH of the casting compound?

For clinical cholangitis to occur two factors must be present: 1) increased intrabiliary pressure and 2) bacteria. The presence of bacteria in bile, however, may actually change its chemical character. Bacteria may secrete glycoproteins and enzymes. Bacterial enzymes may deconjugate both bilirubin and bile salts. Deconjugated bile salts are more likely to diffuse into and damage cells and, thereby, increase their permeability. Another interesting study, therefore, would be to add deconjugated bile salts to the casting compound.

Finally, various bacteria may either enhance or impede cholangiovenous or cholangiolymphatic reflux. Possible enhancing mechanisms have been mentioned above. Could the bacterial production of mucus glycoproteins actually block reflux pathways? Alternatively, could the presence of *pili* on certain bacteria promote their attachment to ductal epithelial cells and, thereby, inhibit reflux? Thus, as with many good studies, the work of Stewart *et al*[4] may have raised more questions than were answered. Hopefully, future studies on the pathogenesis of cholangitis will address 1) the rate of pressure rise, 2) the influence of species difference, 3) the impact of bile composition, and 4) variable effects of different bacteria.

**Keywords:** Corrosion cast, cholangiovenous reflux, cholangitis.

Henry A. Pitt
Blalock 688
Johns Hopkins Hospital
600 N. Wolfe Street
BALTIMORE, MD 21205
USA

**REFERENCES**

1. Mixter, H.W., Rigler, L.G., and Gonzales-Oddone, M.V. (1947) Experimental studies on biliary regurgitation during cholangiography. *Gastroenterology,* **9**, 64–80
2. Huang, T., Bass, J.A. and Williams, R.D. (1969) The significance of biliary pressure in cholangitis. *Archives of Surgery,* **98**, 629–32
3. Yamamoto, K. and Phillips, M.J. (1984) A hitherto unrecognized bile ductular plexis in normal rat liver *Hepatology,* **4**, 381–85
4. Stewart, L., Pellegrini, C.A. and Way, L.W. (1988) Cholangiovenous reflux pathways as defined by corrosion casting and scanning electron microscopy. *American Journal of Surgery,* **155**, 23–27

